# Single Amino Acid Substitutions in the Chemotactic Sequence of Urokinase Receptor Modulate Cell Migration and Invasion

**DOI:** 10.1371/journal.pone.0044806

**Published:** 2012-09-25

**Authors:** Katia Bifulco, Immacolata Longanesi-Cattani, Paola Franco, Vincenzo Pavone, Pietro Mugione, Gioconda Di Carluccio, Maria Teresa Masucci, Claudio Arra, Giuseppe Pirozzi, Maria Patrizia Stoppelli, Maria Vincenza Carriero

**Affiliations:** 1 Department of Experimental Oncology, National Cancer Institute of Naples, Naples, Italy; 2 Institute of Genetics and Biophysics “Adriano Buzzati-Traverso”, National Research Council, Naples, Italy; 3 Department of Chemical Sciences, University of Naples “Federico II”, Naples, Italy; City of Hope National Medical Center and Beckman Research Institute, United States of America

## Abstract

The receptor for urokinase-type plasminogen activator (uPAR) plays an important role in controlling cell migration. uPAR binds urokinase and vitronectin extracellular ligands, and signals in complex with transmembrane receptors such as Formyl-peptide Receptors (FPR)s and integrins. Previous work from this laboratory has shown that synthetic peptides, corresponding to the uPAR_88–92_ chemotactic sequence, when carrying the S90P or S90E substitutions, up- or down-regulate cell migration, respectively. To gain mechanistic insights into these opposite cell responses, the functional consequences of S90P and S90E mutations in full-length uPAR were evaluated. First, (HEK)-293 embryonic kidney cells expressing uPAR^S90P^ exhibit enhanced FPR activation, increased random and directional cell migration, long-lasting Akt phosphorylation, and increased adhesion to vitronectin, as well as uPAR/vitronectin receptor association. In contrast, the S90E substitution prevents agonist-triggered FPR activation and internalization, decreases binding and adhesion to vitronectin, and inhibits uPAR/vitronectin receptor association. Also, 293/uPAR^S90P^ cells appear quite elongated and their cytoskeleton well organized, whereas 293/uPAR^S90E^ cells assume a large flattened morphology, with random orientation of actin filaments. Interestingly, when HT1080 cells co-express wild type uPAR with uPAR S90E, the latter behaves as a dominant-negative, impairing uPAR-mediated signaling and reducing cell wound repair as well as lung metastasis in nude mice. In contrast, signaling, wound repair and in vivo lung metastasis of HT1080 cells bearing wild type uPAR are enhanced when they co-express uPAR^S90P^. In conclusion, our findings indicate that Ser^90^ is a critical residue for uPAR signaling and that the S90P and S90E exert opposite effects on uPAR activities. These findings may be accommodated in a molecular model, in which uPAR^S90E^ and uPAR^S90P^ are forced into inactive and active forms, respectively, suggesting important implications for the development of novel drugs targeting uPAR function.

## Introduction

Cell migration is important during normal development and tissue repair, and requires a coordinated regulation of extracellular matrix proteolysis, adhesion, and signaling [1]. Its dysregulation underlies numerous disorders such as chronic inflammation, vascular disease and tumor metastasis [2].

The receptor for urokinase-type plasminogen activator (uPAR) plays an important role in controlling cell migration [3], [4]. uPAR is a glycosylated glycosyl-phosphatidyl-inositol (GPI)-anchored protein [5] formed by three domains (DI, DII, and DIII) connected by short linker regions [6]. Besides being responsible for focalizing uPA-mediated plasminogen activation on cell surfaces [7]–[8], uPAR also promotes intracellular signalling, thus regulating physiological processes such as wound healing, immune responses and stem cell mobilization, as well as pathological conditions such as inflammation and tumor progression [9]–[12]. Consistent with its multifunctional role, uPAR binds the extracellular ligands uPA and vitronectin (Vn) and cooperates with transmembrane receptors such as Formyl-peptide Receptors (FPR)s and integrins [1], [13]. Biochemical and cellular evidence shows that uPA binding modulates the interaction between uPAR and Vn, both at the biochemical and the cellular level [14]–[16]. The uPAR/Vn interaction stimulates signaling, leading to cytoskeletal rearrangements and cell migration [14]–[17]. The link between the uPA/uPAR system and Vn receptors (VnR)s is further supported by the ability of uPA to directly interact with αvβ5 VnR, suggesting a bridging of uPAR and αvβ5 mediated by uPA [18].

Membrane-associated and soluble forms of uPAR, containing the ^88^Ser-Arg-Ser-Arg-Tyr^92^ sequence (uPAR_88–92_) connecting DI and DII domains, as well as the synthetic peptide SRSRY are able to trigger *in vitro* and *in vivo* cell migration and angiogenesis [19]–[22]. The uPAR_88–92_ sequence interacts with FPRs type 1 and 2, thus inducing cell migration [11], [19]–[23], in an integrin-dependent manner [23]. Furthermore, upon binding to FPR, the synthetic peptide SRSRY causes FPR internalization and triggers VnR activation with an inside-out type of mechanism [21]–[22]. Ala-scan studies indicated that the Arg^91^ and Tyr^92^ residues in the DI–DII linker are essential for cell morphological changes [24] and are crucial residues for binding to the N-terminal somatomedin B domain of Vn, shedding light on the uPAR structure-function relationship [25]–[26]. We have also found that the Arg^89^-Ser-Arg^91^ central core is of particular interest for the SRSRY-dependent cell signaling [27], by studying SRSRY peptide analogues. In an attempt to specifically inhibit the uPAR_88–92_ signaling, we have found that penta- and tetra peptides carrying a Ser^90^ to Glu substitution inhibit SRSRY-, fMLF- and serum-directed cell migration [27]–[28], whereas peptides carrying a Ser^90^ to Pro substitution exhibit a higher chemotactic activity than SRSRY (Pavone and Carriero, unpublished).

Emerging evidence shows that some of the uPAR functional effects are supported by conformational changes of the receptor: a few years ago, Yuan and Huang have suggested that unengaged uPAR may exist in a latent, inactive form which may be activated by an uPA-dependent conformational change [29]. More recently, Gardsvoll et al. have proposed that there is a large conformational flexibility in the assembly of the three uPAR domains, so that the receptor may acquire different conformational states. According to this model, upon uPA engagement, uPAR switches from an open to an intermediated and then to a closed conformation which differently regulates formation of lamellipodia on Vn-coated surfaces [30]. Analysis of the most frequently observed conformations of the sequence Arg-X_1_-Arg-X_2_ (X_1_: any amino acid; X_2_: Tyr, Phe, Trp), which is related to uPAR_89–92_ sequence, in a PDB [31] data set of 406 protein structures, revealed that Arg-Ser-Arg-X_2_ sequence shows an equal distribution among the α-turned, β-extended, or random conformation, whereas the Arg-Glu-Arg-X_2_ is mainly observed in α-turn conformation, and Arg-Pro-Arg-X_2_ is mainly observed in a β-extended conformation [28]. In this work, by extending our previous information on the 3-D structure of the core peptide RSRY [28], we have studied the conformational preferences of the uPAR_89–92_ sequence in the published x-ray structures of SuPAR [32]–[34]. We confirm that at least two different conformations may be adopted by the uPAR_89–92_ region, possibly reflecting the latent inactive *versus* ligand-activated receptor. In this view, Ser^90^ appears as a crucial residue which may affect the conformation of the receptor chemotactic region. To address whether mutations of Ser^90^ may affect receptor function, we have analyzed the biological properties of full length, membrane-associated uPAR carrying Ser^90^ substituted with a Glu, or a Pro residue. Here, we provide evidence that expression of uPAR^S90P^ increases cell adhesion to Vn, migration toward ATF or fMLF, enhances agonist-triggered FPR internalization and increases *in vitro* and in *vivo* cell migration and invasion. In contrast, cells bearing uPAR^S90E^ exhibit a reduced binding and adhesion to Vn, an impaired agonist-induced FPR internalization and a dramatic reduction of *in vitro* and in *vivo* cell migration and invasion. Finally, co-expression of uPAR^S90E^ with endogenous uPAR in the HT1080 fibrosarcoma cells injected into mouse tail vein, dramatically reduces lung metastasis, indicating the occurrence of a clear-cut dominant-negative effect of the uPAR^S90E^ variant.

## Results

### Opposite regulation of 293 cell responses by uPAR^S90E^ or uPAR^S90P^ variants

**Figure 1:**
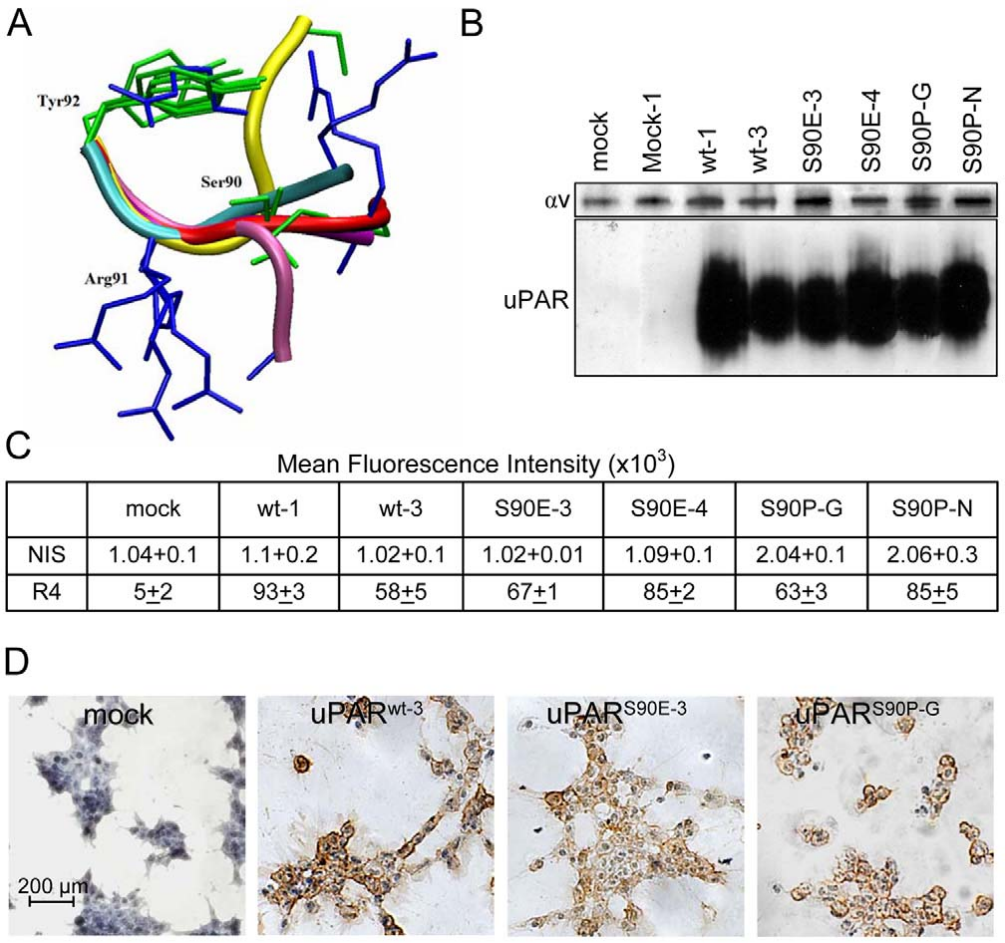
uPAR carrying S90E and S90P substitutions. A : Arg^91^ and Tyr^92^ superposition of the backbone atoms of the various 88–92 segments of SuPAR from x-ray structures. Residue 89 is not visible in the electron density map of 1YWH. Side chains are reported as stick, Arg residues are in blue. Backbones are reported as ribbon drawing: Yellow, 2I9B, chain E and F, residues 88–92; Red, 3BT2, chain U, residues 88–92; Mauve, 1YWH, chain E, residues 89–92; Cyan, 1YWH chain M, residues 89–92; Purple, 1YWH chain A, residues 89–92. **B**. Lysates (25 µg/sample) from 293 stably transfected either with pcDNA3 empty vector (293/mock) or pcDNA3 coding for human uPAR (293/uPAR^wt^, uPAR^S90E^, uPAR^S90P^), were resolved on a 10% SDS-PAGE followed by Western blotting with R4 anti-uPAR or anti-αv mAbs. **C**. Cytofluorimetric analysis of the indicated stably transfected 293 clones with R4 anti-uPAR mAb or nonimmune immunoglobulins. **D**. Representative images of the indicated stably transfected 293 clones immuno-stained with anti-uPAR R4 mAb.

Previous work from this laboratory has shown that tetra- and penta-peptides derived from the uPAR_88–92_ chemotactic sequence (SRSRY) and carrying specific substitutions of Ser^90^ modulate *in vitro* and *in vivo* tumor cell migration, raising the hypothesis that mutations in Ser^90^ may affect receptor conformation and function [27]–[28]. Therefore, we first investigated the conformational preferences of the SRSRY sequence in the x-ray structures of SuPAR (Protein Data Bank code, 1YWH, 2I9B, 3BT2) [32]–[34]. We found that the linker Cys76-Cys95 that includes the SRSRY sequence, is quite flexible and largely undetermined in the electron density map. Fig. 1A reports the superposition of the backbone atoms of Arg^91^ and Tyr^92^ of the various 88–92 segments of SuPAR structures, whenever visible in the electron density map. The Arg-Ser-Arg-Tyr sequence adopts either α turned (2I9B) or β-extended (3BT2, 1YWH) conformation and Ser^90^ appears as a critical hinge residue to control the shift between these different conformations. This observation is in agreement with previous findings on a larger ensemble of proteins containing the Arg-Ser-Arg-Tyr sequence, which adopts either a random, α-turned or β-extended conformation, with a quite equal distribution among the three classes of conformations [28]. The fact that the Arg-Glu-Arg-Tyr sequence is mainly observed in an α-turn conformation, whereas the Arg-Pro-Arg-Tyr is mainly observed in the β-extended conformation, suggests that in uPAR the Arg-Glu-Arg sequence may favour a turned structure of the whole chemotactic region, while the Arg-Pro-Arg sequence may favour an extended conformation, respectively. These differences might be associated to inhibitory or stimulatory functional properties. Therefore, the open form could be favoured in the uPAR^S90E^ variant, while the closed form could be favoured in the uPAR^S90P^ variants. To test whether our prediction might be true in the context of full-length receptor, two variants carrying S90E and S90P substitutions of Ser^90^ were generated and tested for their effects on receptor ability to regulate cell adhesion, migration and invasion. 293 cells were chosen because they express neither uPA nor uPAR [24]. The expression level of uPAR (293/uPAR^wt^), uPAR^S90E^ or uPAR^S90P^ mutants in 293 stable transfectants was analyzed by Western blot and FACS analysis using R4 monoclonal antibody which recognizes an epitope located on uPAR DIII not involving mobile domain interfaces (Fig. 1B–C). Clones exhibiting equivalent receptor expression for the wild-type and mutated forms of uPAR (293/uPAR^wt-3^, uPAR^S90E-3^, and uPAR^S90P-G^) were selected for further analyses (Fig. 1B–C). Immunocytochemical staining with R4 mAb confirmed that, unlike 293/mock cells, all clones express uPARs on cell surfaces at a similar extent (Fig. 1D).

**Figure 2:**
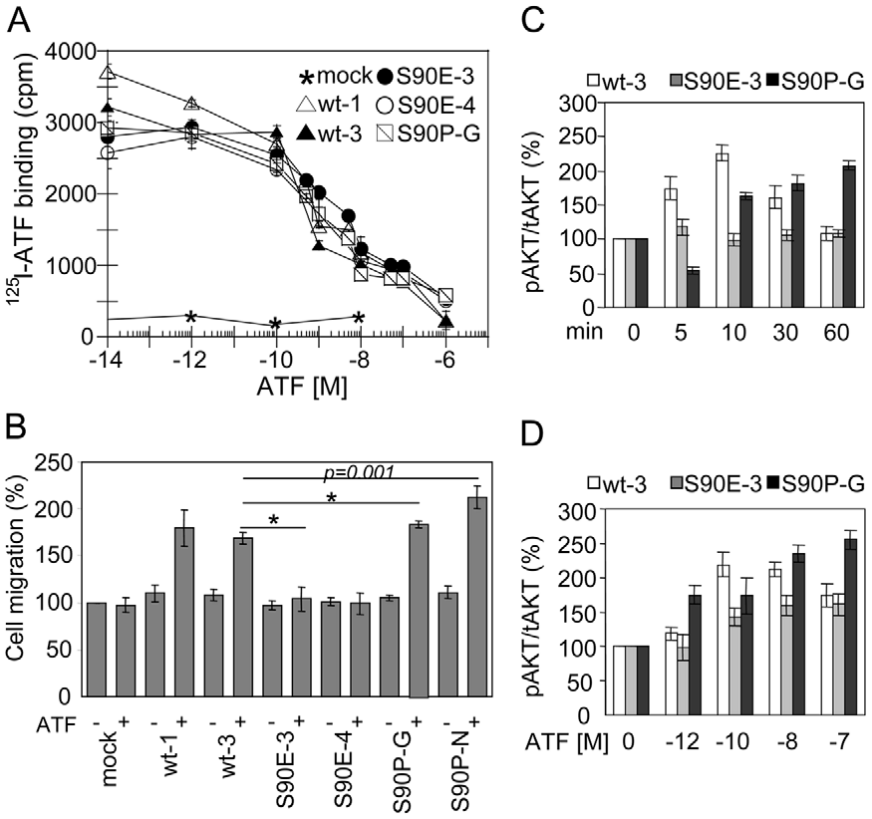
uPAR^S90E^ and uPAR^S90P^ retain the ability to bind to ATF. A . Transfected 293 clones were grown adherent (2×10^5^cells/well) on 24-multiwell plates. ^125^I-ATF (150,000 cpm/sample) was incubated with cells, in the presence of increasing concentrations of unlabeled ATF for 3 hours at 4°C and cell surface-associated radioactivity was determined. Data represent the mean of specific binding ± SD of three independent experiments performed in duplicate. **B**. Directional cell migration of the indicated transfected 293 clones toward 10 nM ATF. The extent of migration is expressed as percentage of cell migration of 293/mock cells assessed in the absence of ATF, considered as 100%. Data represent the mean ± SD of three independent experiments in triplicate. *: *p*<0.001. **C–D**. Whole cell lysates(50 µg/sample) from cells exposed to ATF for the indicated times (C) or concentrations (D) immunoblotted with anti-phospho-AKT Ab (pAKT) and then with anti-Akt mAb (tAKT). Quantitative assessment of the pAKT and tAKT content of each sample was performed using NIH Image 1.62 software. Data are means of three experiments, with SD indicated by error bars.

Unlike 293/mock cells, all transfectants bind to ATF. The B_max_ calculation revealed a comparable receptor density for 293/uPAR^wt-3^, uPAR^S90E-3^, and uPAR^S90P-G^ cell clones (∼3.14×10^5^, ∼3.74×10^5^ and ∼3.42×10^5^ uPARs/cell, respectively) and a slightly higher receptor number for 293/uPAR^wt-1^ and uPAR^S90E-4^ cells (∼3.82×10^5^ and 4.13×10^5^ uPARs/cell, respectively). Furthermore, 293/uPAR^wt-3^ and 293/uPAR^S90P-G^ cells bind to ATF with a similar Kd_app_ (∼1 nM and 0.7 nM, respectively), whereas the Kd_app_ of both 293/uPAR^S90E^ clones appeared to be 10-fold higher (Fig. 2A). These data indicate that the S90E mutation in the full molecule slightly decreases uPAR affinity for ATF. Because this mutation does not involve a residue directly engaged in ligand binding [35], this finding raises the possibility that the S90E supports a conformational change of the linker domain. As a consequence, changes in the orientation and/or mobility of the DI domain relative to DII and DIII domains might impact on ATF binding. Next, we explored the ability of 293/uPAR^S90E^ and 293/uPAR^S90P^ clones to respond to 10 nM ATF in a standard Boyden chamber assay. As expected, 293/mock cells failed to respond to ATF, whereas cells expressing wild type uPAR (293/uPAR^wt-1^, 293/uPAR^wt-3^), as well as 293/uPAR^S90P^ (293/uPAR^S90P-G^, 293/uPAR^S90P-N^) clones did migrate toward ATF (Fig. 2B). Interestingly, both 293/uPAR^S90E-3^ and 293/uPAR^S90E-4^ clones did not migrate toward ATF, even at 100 nM (105+/−6% and 110+/−8% of the basal cell migration assessed in the absence of any chemoattractant, respectively). Ligand-activated uPAR triggers an intracellular signal that activates PI3K/Akt pathway [36]. As shown in Fig. 2C, cell exposure to 10 nM ATF induced a time dependent AKT activation in 293/uPAR^wt-3^ and 293/uPAR^S90P-G^ cells. Interestingly, a long-lasting Akt phosphorylation was observed in 293/uPAR^S90P-G^ as compared to 293/uPAR^wt-3^ cells. Unlike 293/uPAR^S90E-3^, 293/uPAR^S90P-G^ and uPAR^wt-3^ cells exhibit a clear-cut dose-dependent increase in Akt phosphorylation (Fig. 2D). In conclusion, these experiments indicate that uPAR in which the Ser^90^ is substituted with a glutamic acid residue prevents receptor signaling and slightly reduces ATF/uPAR interaction.

### Expression of uPAR^S90E^ or uPAR^S90P^ variants induces changes in 293 cell morphology, cytoskeletal organization and migration

**Figure 3:**
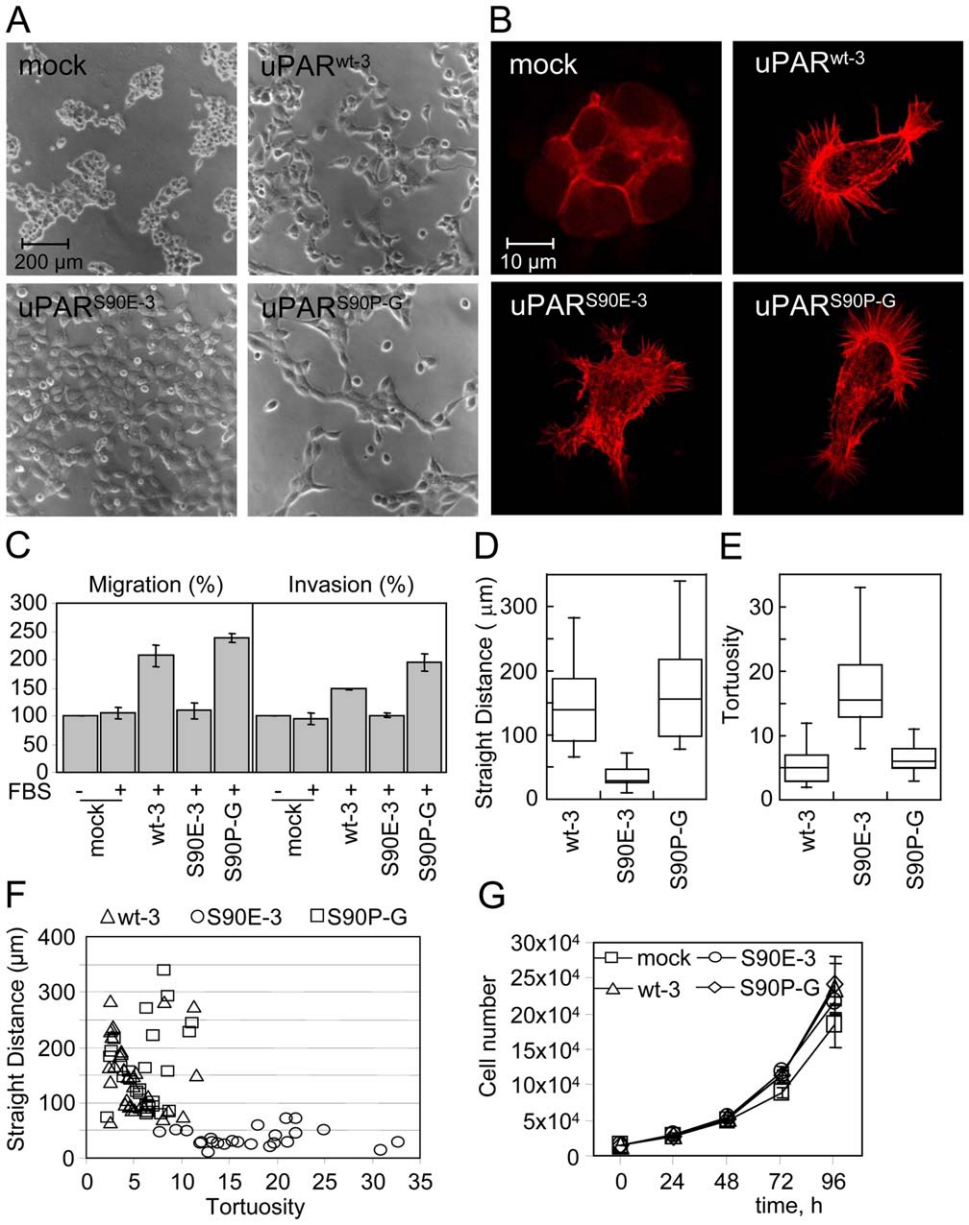
Expression of uPAR^S90E^ and uPAR^S90P^ induces changes in 293 cell morphology, cytoskeletal organization and migration without affecting cell proliferation. A–B . Representative images of the indicated transfected 293 clones analyzed by phase contrast microscopy (A) or stained with rhodamine-phalloidin (B). **C**. Cell migration and invasion of stably transfected 293 cells toward 10%FBS. The extent of migration or invasion is expressed as percentage of migrating or invading 293/mock cells in the absence of chemoattractant, considered as 100% (none). Data represent the means ± SD of three experiments in duplicate. **D–E**. Cell tracking analysis of the steady-state transfected 293 cells. Cells were recorded for 70 minutes every 15 sec. while kept at 37°C, under a 5% CO2 atmosphere. Ten cells/field were followed in a total of 3 independent experiments. Straight distance (D) and tortuosity (E) were quantified using the Axiovision 4.8 software (Carl Zeiss). In F straight distance versus tortuosity is plotted for each cell analyzed. G. Time-dependent proliferation of transfected 293 cells in growth medium. Data represent the means ± SD of three experiments, performed in duplicate.

Similarly to wild type, 293/mock cells exhibit an epithelial morphology with a circumferential ruffled margin. In agreement with the uPAR-dependent morphological changes reported by Madsen et al [24], the expression of human uPAR induces changes in cell morphology, including a reduced cell-cell contact and the formation of extensive lamellipodia. These changes are observed also in 293/uPAR^S90P-G^ cells that assume a more elongated morphology. Instead, 293/uPAR^S90E-3^ cells appear closely adherent along their lateral and apical surfaces and assume a larger, flattened morphology and the loss of polarity (Fig. 3A). Changes in cell morphology reflect the reorganization of the F-actin cytoskeleton. In particular, a random orientation of actin filaments was observed in 293/uPAR^S90E-3^ cells (Fig. 3B).

To explore whether uPA-independent, general mechanisms underlying migration were affected in 293 bearing mutated receptors, analysis of cell migration was conducted in Boyden chambers toward FBS for 4 hours. Data show that 293 cells expressing uPAR^wt^ or uPAR^S90P^ efficiently migrate toward 10% FBS (207%+/−19% and 239%+/−8% of the basal cell migration, respectively), whereas 293 cells expressing uPAR^S90E^ do not, showing that the S90E mutation impairs general cell motility (Fig. 3C). Interestingly, analysis of basal cell motility using time-lapse microscopy reveals that cell movements of both 293/uPAR^wt-3^ and 293/uPAR^S90P-G^ cells are more straight-line (153+/−64 µm/70 minutes and 163+/−72 µm/70 minutes, respectively) and less tortuous (5+/−2 and 6+/−2, respectively) as compared with the straight distance and tortuosity of 293/uPAR^S90E-3^ cells (35+/−16 µm/70 minutes and 17+/−6, respectively) (Fig. 3D–E–F and movies in Fig. S1, S2 and S3). To ascertain if the observed impairment in migration may affect invasion, the ability of transfected clones to cross matrigel was analyzed. As expected, 293/mock cells did not cross matrigel toward FBS, while 293/uPAR^wt-3^ cells, and more appreciably 293/uPAR^S90P-G^ cells invade matrigel (148%+/−1% and 194+/−15% of the basal cell invasion, respectively). On the contrary, 293/uPAR^S90E-3^ cells behave as 293/mock cells (Fig. 3C). These effects on invasion are not due to increased proliferation, as neither 293/uPAR^S90P-G^ nor 293/uPAR^S90E-3^ cells exhibit any difference in the proliferation rate. As shown in Fig. 3G, number of cells observed over a period of 96 hours was similar, regardless expression of wild type or mutant receptors (p>0.05). All together, these findings indicate that Ser^90^ is crucial to uPAR-dependent signaling and that the S90E substitution dramatically affects cell morphology, cytoskeletal organization, migration and invasion.

### Impact of S90E and S90P mutations on the uPAR ability to trigger FPR activation and internalization

**Figure 4:**
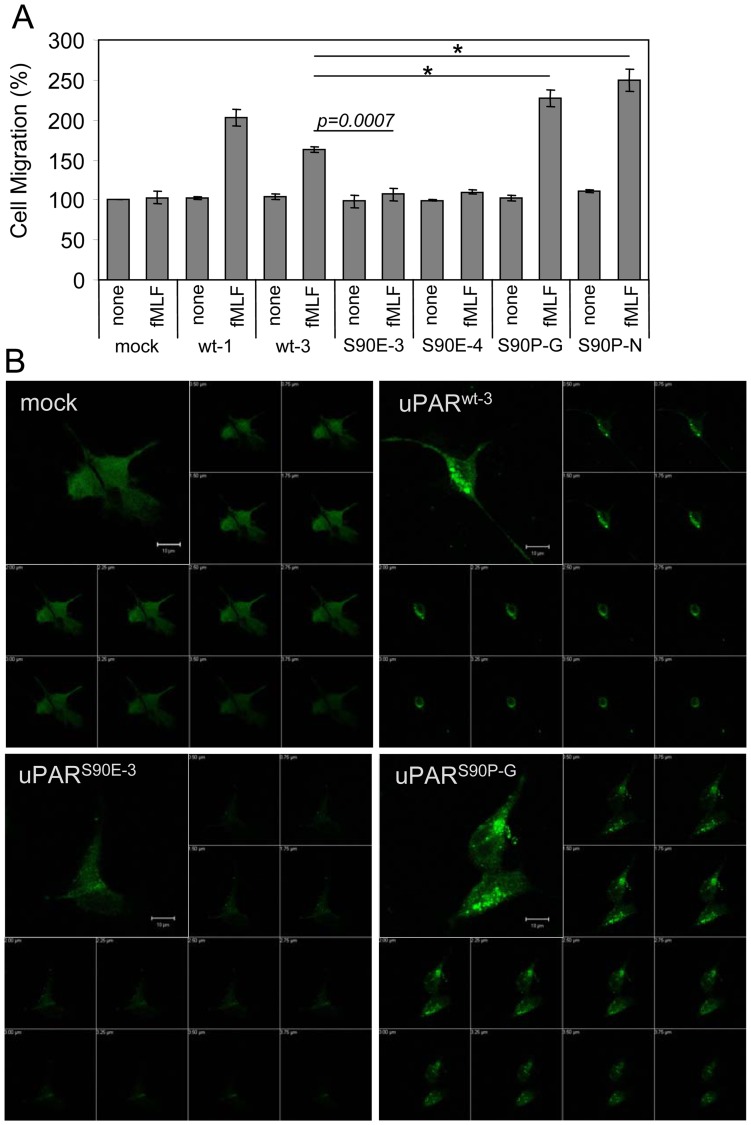
Expression of uPAR^S90E^ or uPAR^S90P^ oppositely modulates FPR activation and internalization. A . Cell migration of transfected 293 cells toward 10 nM fMLF. The extent of migration is expressed as percentage of cell migration of 293/mock cells assessed in the absence of fMLF, considered as 100% (none). Data represent the means ± SD of three independent experiments in triplicate. *: *p*<0,0001. **B**. Agonist-triggered FPR internalization in transfected 293 cells. Cells grown adherent on glass slides to semi-confluence were incubated with 10 nM N-formyl-Nle-Leu-Phe-Nle-Tyr-Lys-fluorescein for 30 minutes at 37°C and then visualized using a Zeiss 510META LSM microscope. Z-series images represent focal planes corresponding to 0.25 μm vertical interval. Scale bar10 µm. Original magnifications: 630x.

We and others have previously documented that: *i*) uPAR binds to FPRs through its Ser^88^-Arg-Ser-Arg-Tyr^92^ sequence, thus promoting dose-dependent directional cell migration; *ii*) cell desensitization with an excess of fMLF abrogates uPAR-dependent FPR activation; *iii*) soluble forms of uPAR containing the Ser^88^-Arg-Ser-Arg-Tyr^92^ sequence or the synthetic peptide SRSRY activate FPR, favouring its internalization in endothelial cells [11], [20]–[22]. To investigate the effects of S90E and S90P mutations on uPAR ability to trigger FPR activation, the ability of transfected clones to migrate toward *N*-formyl-methionyl-leucyl-phenylalanine (fMLF) was assessed. As wild type 293 cells which express FPR but do not migrate toward fMLF [20]–[22], [37], [38], 293/mock cells failed to respond to fMLF. In contrast, 10 nM fMLF elicits a considerable response of 293/uPAR^wt-3^ and 293/uPAR^S90P-G^ as well as 293/uPAR^S90P-N^ cells, reaching 163%, 227% and 249% of the basal cell migration, respectively. On the other hand, fMLF failed to elicit migration of 293/uPAR^S90E-3^ and 293/uPAR^S90E-4^ cells that behave as 293/mock (Fig. 4A). Overall, these findings suggest that fMLF-triggered FPR activation is enhanced in 293/uPAR^S90P^cells and is abrogated in cells bearing uPAR^S90E^. In response to agonist-stimulation, FPRs are internalized and the uPAR_88-92_ sequence itself favours FPR internalization [27]–[28]. To evaluate the effect of S90E and S90P mutations on uPAR-dependent FPR internalization, binding experiments were performed. Cells exposed to 10 nM *N*-formyl-Nle-Leu-Phe-Nle-Tyr-Lys-fluorescein (fMLF-fluorescein) at 37°C, were analysed by a confocal microscope. As expected, FPR probed by its fluorescent agonist appeared to be mainly localized on 293/mock cell surface whereas in 293/uPAR^wt-3^ cells it appeared in intra-cytoplasmic green fluorescent spots (Fig. 4B) which were undetectable in cells pre-incubated with an excess of non-fluorescent fMLF (not shown). Upon 293/uPAR^S90P-G^ cell exposure to fluorescent agonist, FPR appeared more efficiently internalized in the 75% cell population as confirmed by z-stack analysis of confocal images (Fig. 4B). Interestingly, agonist-dependent FPR internalization was strongly reduced in cells expressing uPAR^S90E^, confirming that uPAR signaling involving FPR is impaired, in the presence of the S90E mutation.

### Effect of S90E and S90P mutations on uPAR association with vitronectin and vitronectin receptor

**Figure 5:**
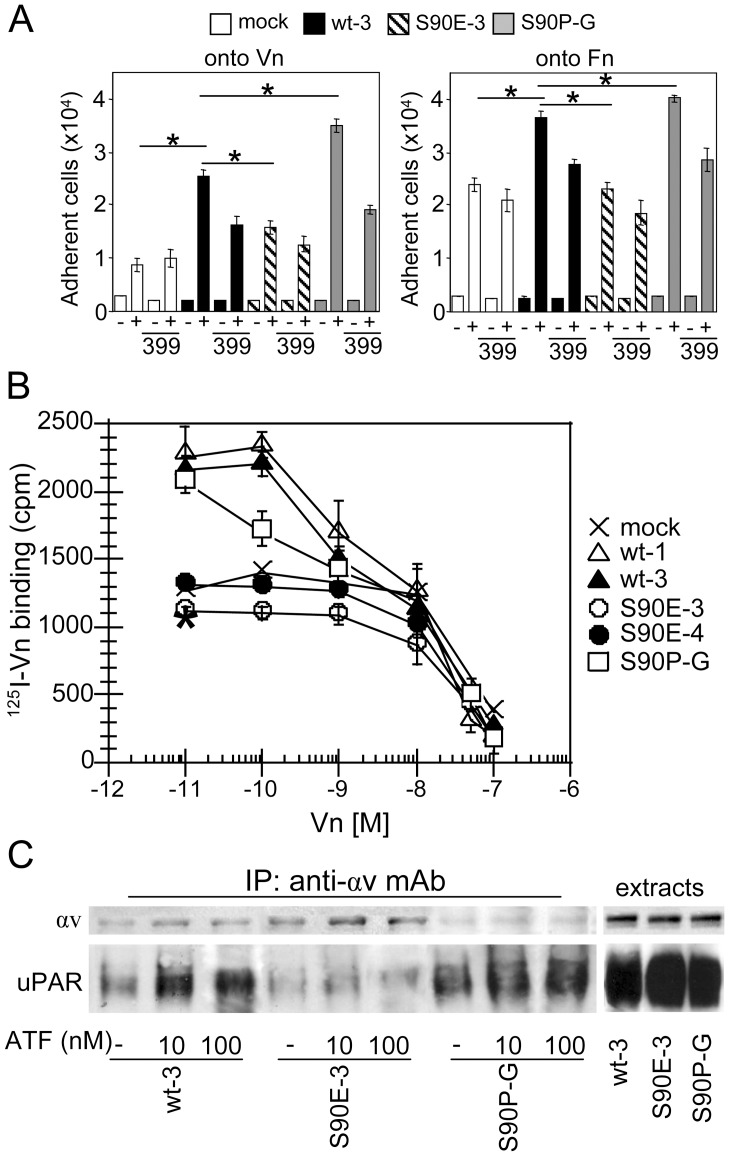
S90E and S90P substitutions affect uPAR/vitronectin and uPA-dependent uPAR/vitronectin receptor association. A . Transfected 293 cells (1×10^5^/well) were pre-incubated for 1 hour with 2 µg/ml 399 anti-uPAR Ab or diluents and allowed to adhere for 1 hour in 24-well dishes coated with BSA (−), Vn or Fn (+). Data are reported as number of adherent cells and represent the average of three different experiments performed in duplicate. * *p*<0.0001. **B**. Transfected 293 clones were grown adherent (2×10^5^cells/well) on 24-multiwell plates. ^125^I-Vn (150,000 cpm/sample) was added in the presence of increasing concentrations of unlabeled Vn for 3 hours at 4°C and cell surface-associated radioactivity was determined. Data represent the mean of specific binding ± SD of three independent experiments in duplicate. * Cell associated radioactivity of 293/uPAR^wt-3^ cell-pre-exposed to P1F6 anti-αvβ5 mAb. **C.** Lysates (400 µg/sample) from the indicated transfected 293 cells exposed for 60 minutes to 10 nM ATF, 100 nM ATF or diluents (-), were immunoprecipitated with 5 µg/ml anti-αv Ab. The resulting proteins were analyzed by Western blot using R4 anti-uPAR or anti-αv mAbs. Right: 50 µg of the indicated transfected 293 cells were loaded as control.

We explored the possibility that S90P and S90E substitutions may affect uPAR-dependent cell adhesion onto Vn. As expected, mock-transfected 293 cells which express αvβ5 integrin on their surface [21], moderately adhere to Vn and the expression of uPAR increases their adhesion to Vn (Fig. 5A). Interestingly, the expression of uPAR^S90P^ further increases cell adhesion, whereas 293/uPAR^S90E-3^ cells adhere to Vn less than 293/uPAR^wt-3^. The observed differences in the extent of adhesion are uPAR-dependent because they were reduced by cell pre-exposure to 399 anti-uPAR polyclonal Abs (Fig. 5A). To further test the impact of Ser^90^ mutations on the affinity of the uPAR/Vn interaction, competition assays with ^125^I-Vn/unlabeled Vn on intact 293 transfectants were performed. Mock-transfected 293 cells specifically bound to Vn with a Kd_app_ ∼50 nM whereas uPAR expressing 293 cells exhibited a 42% increased specific association of ^125^I-Vn to cell surface (Kd_app_ ∼10 nM) due to the co-expression of uPAR and αvβ5 integrin. In fact, a 46% of binding reduction was observed by 293/uPAR^wt-3^ cell-pre-exposure to the anti-RGD-binding site P1F6 mAb, highlighting the contribution of integrin to the binding to Vn (*, Fig. 5B). In 293/uPAR^S90P-G^ cells 50% competition was achieved at ∼10 nM, whereas 293/uPAR^S90E-3^ cells exhibit a reduced affinity to Vn, as 50% competition was achieved at ∼50 nM (Fig. 5B). All together these findings indicate that uPAR^S90P^ retains the ability to bind to Vn, which is abrogated by the S90E mutation. To investigate whether the effects of S90E and S90P substitutions on cell adhesion are exclusively due to a specific impairment of uPAR/Vn interaction, cell adhesion onto fibronectin (Fn) was tested under the same conditions. As shown in Fig. 5A, right panel, cells carrying uPAR^S90E^ or uPAR^S90P^ exhibit a decreased or increased adhesion on Fn, respectively. Again, the differences in adhesion were reduced by cell pre-exposure to 399 anti-uPAR polyclonal Abs. These findings raise the possibility that, besides the direct modulation of uPAR/Vn binding, more complex mechanisms involving a cross-talk between uPAR and integrins may occur.

Although a direct physical interaction between uPAR and integrins has been not really proved [1], a variety of conditions, including cell exposure to uPA, modulates the formation of uPAR/integrin complexes. To assess if the S90E or S90P point mutations affect uPAR/αv integrin association, co-immunoprecipitation assays were performed on transfected cells pre-exposed to ATF. As shown in Fig. 5C, an appreciable amount of uPAR co-purified with αv was observed in 293 cells bearing wild type or mutated S90P forms of uPAR which increases upon cell exposure to 10 nM or 100 nM ATF for 60 minutes. In contrast, the extent of αv co-purified with uPAR in an ATF-dependent manner was reduced in cells bearing uPAR^S90E^ and 100 nM ATF still failed to promote uPAR/αv association. Overall, these results indicate that uPAR^S90P^ retains the ability to interact with Vn and VnR, whereas the S90E mutation abrogates uPAR/Vn interaction and uPAR/αv integrin association.

### Impact of S90E and S90P mutations on HT1080 cell invasion *in vitro* and *in vivo*

**Figure 6:**
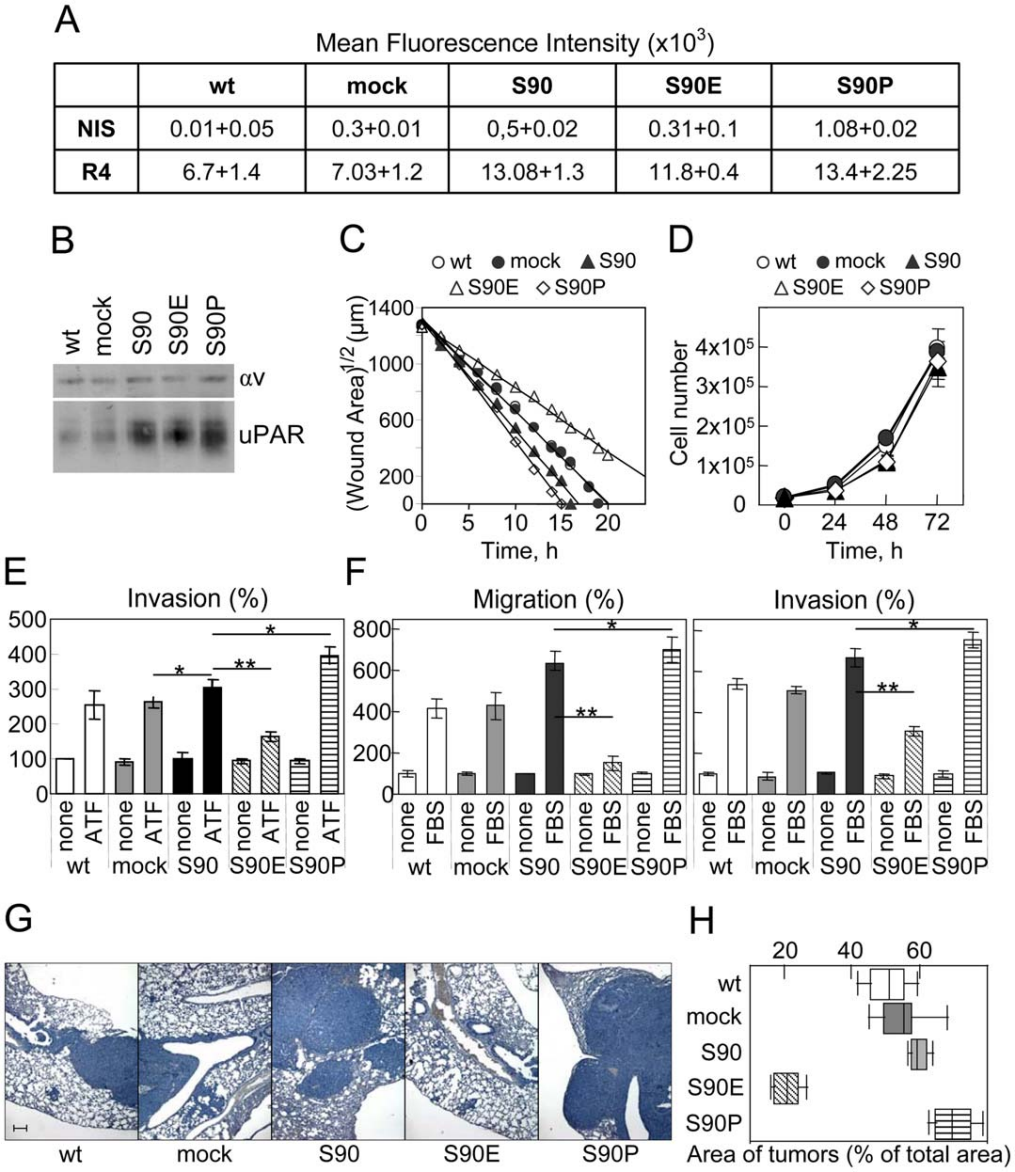
Expression of uPAR carrying S90E and S90P substitutions oppositely regulates HT1080 fibrosarcoma cell migration, invasion and lung colonization of nude mice. HT1080 cells were stably transfected with pcDNA3 empty vector (HT1080/mock) or pcDNA3 loaded with wild type uPAR (uPAR^S90^), uPAR^S90E^ or uPAR^S90P^ cDNAs. **A**. Cytofluorimetric analysis of parental and transfected HT1080 cells with R4 anti-uPAR mAb or non immune immunoglobulins. **B.** 25 µg of membrane extracts from the indicated cells were resolved on a 10% SDS-PAGE followed by Western blotting with R4 anti-uPAR or anti-αv mAbs. **C**. Wound healing of the indicated cells kept in growth medium at 37°C in a 5% CO_2_ of a Zeiss inverted microscope equipped with a motorized stage. One field that includes the scratched path from each dish was selected and scanned sequentially every 30 minutes for 20 hours. The extent of wounded areas was evaluated by the Axiovision 4.8 software and plotted against time. Data points were fitted with a linear equation whose slope represents the cell speed. **D.** Time-dependent proliferation of wild type and the indicated transfected HT1080 cells in growth medium. Data represent the mean of two experiments, performed in triplicate. **E**. Cell invasion of wild type and transfected HT1080 cells toward 10 nM ATF. The extent of invasion is expressed as percentage of invading cells assessed in the absence of ATF, considered as 100% (none). **F.** Cell migration or invasion of the indicated cells toward 10%FBS. The extent of migration or invasion is expressed as percentage of parental migrating or invading HT1080 cells assessed in the absence of FBS, considered as 100% (none). **G–H**. Parental or transfected HT1080 cells (1.5×10^6^ cells as a single-cell suspension in 100 µl of sterile PBS) were injected in the tail vein of thirty (6 animals/group) nude mice. After 22 days, mice were sacrificed and lungs were fixed in buffered 4% formaldehyde for the histological analysis. Representative images of haematoxilin-stained sections derived from the lung of mice injected with wild type or the indicated transfected HT1080 cells. 5x magnification. Scale bar = 200 µm (G). Area of neoplastic foci was assessed in three not serial haematoxilin-stained lung sections at 5x magnification, using the Axiovision 4.8 software and expressed as percentage of total area measured in each tissue section (H).

To investigate the impact of S90E mutation on tumor cell invasion, in the presence of a wild type receptor, we took advantage of the highly motile and invasive fibrosarcoma HT1080 cells [28]. This cell line is known to express uPARs on cell surface as well as to secrete uPA. For these experiments, HT1080 cells were stably transfected with pcDNA3 vector alone (HT1080/mock), or encoding wild type uPAR (HT1080/uPAR^S90^), uPAR^S90E^ (HT1080/uPAR^S90E^) or uPAR^S90P^ (HT1080/uPAR^S90P^) and G418-resistant cells were amplified. FACS analysis shows that the overall uPAR expression of HT1080/uPAR^S90^, HT1080/uPAR^S90E^ and HT1080/uPAR^S90P^ cells is approximately two times greater than that of parental or mock transfected HT1080 cells (based on the mean fluorescence intensity, Fig. 6A). Western blot analysis with R4 mAb confirmed the two times higher uPAR content in HT1080/uPAR^S90^, HT1080/uPAR^S90E^ and HT1080/uPAR^S90P^ pools, as compared to the parental or mock transfected HT1080 cell line (Fig. 6B). First, we tested whether the expression of uPAR^S90E^ or uPAR^S90P^ modifies HT1080 spreading in a wound healing assay monitored for 20 hours by time-lapse video microscopy. The square root of the measured wound areas were plotted against time. Data points were fitted with a linear equation whose slope represents the cell speed. As shown in Fig. 6C and in Fig. S4–S8, in the presence of 10%FBS, wounds of wild type and mock transfected HT1080 cells disappeared after 18 hours, while wound repair of HT1080/uPAR^S90^ occurs within15 hours. Interestingly, wounds of HT1080/uPAR^S90E^ and HT1080/uPAR^S90P^ cells disappeared within 14 and 20 hours, respectively. These different effects on wound repair are not due to differences in cell proliferation, as shown by assaying the proliferation rate of parental and stably transfected HT1080 (Fig. 6D). To identify the specific contribution of uPAR variants to induce cell invasion, Boyden chamber assays were performed using ATF as chemoattractant. As shown in Fig. 6E, parental as well as mock-transfected HT1080 cells exhibit an appreciable ability to invade matrigel (255+/−39% and 261+/−16% of the basal cell invasion, respectively), slightly lower than HT1080/uPAR^S90^ (304+/−13%), whereas co-expression of uPAR^S90E^ or uPAR^S90P^ strongly decreases (164+/−13%) or increases (394+/−23%), respectively, the extent of ATF-dependent HT1080 cell invasion (Fig. 6E). Similar results were obtained when FBS was employed as a chemoattractant (Fig. 6F). As expected, co-expression of endogenous uPAR with uPAR^S90^ increases the extent of FBS-dependent HT1080 cell migration. Interestingly, co-expression of uPAR^S90E^ or uPAR^S90P^ strongly decreases or increases, respectively, the extent of FBS-dependent HT1080 cell migration and invasion (Fig. 6F). These findings raise the possibility that additional mechanisms may be operating, possibly controlling uPAR downstream effectors.

Finally, the effect of S90E and S90P mutations were investigated *in vivo* in a mouse lung colonization model. Parental HT1080, HT1080/mock, HT1080/uPAR^S90^, HT1080/uPAR^S90E^ or HT1080/uPAR^S90P^ cells (1.5×10^6^) were injected in the tail vein of 30 nude mice. After 22 days, mice were sacrificed, lungs were removed, fixed in buffered 4%formaldehyde and examined blindly. The extent of normal and tumor lung parenchyma was measured in three fields/sample on haematoxilin-stained sections. Area of lung metastasis was assessed using the Axiovision 4.8 software and the results were expressed as percentage of total area measured in each tissue section. Morphometric analysis of neoplastic foci revealed a mean neoplastic area significantly higher in mice injected with HT1080/uPAR^S90^ cells (60+/−3%, of total area measured in the tissue section), as compared with those injected with parental or mock transfected HT1080 cells (51+/−7% and 56+/−8%, respectively, in both cases: *p*<0.001). Moreover, a mean neoplastic area significantly higher was found in lung sections of mice injected with HT1080/uPAR^S90P^ cells (71+/−6%; HT1080/uPAR^S90P^ versus HT1080^S90^: *p*<0.001) whereas it was significantly lower in mice injected with HT1080/uPAR^S90E^ (19+/−8%; HT1080/uPAR^S90E^ versus HT1080^S90^ *p*<0.0001) (Fig. 6G and H).

## Discussion

There is an overwhelming evidence that uPAR plays a key role in pathological processes sustained by an altered cell migration such as angiogenesis, tumor invasion, inflammation and mobilization of haematopoietic stem cells. Therefore, unraveling structural requirements modulating uPAR function is a prerequisite to develop new receptor antagonists as promising anti-metastatic and/or anti-inflammatory drugs. In this paper, we present evidence that the substitution of Ser^90^ in the uPAR chemotactic sequence with a proline residue enhances agonist-triggered FPR activation and internalization, increases cell adhesion onto Vn and favors uPAR/VnR association. In contrast, the substitution of Ser^90^ with a glutamic acid residue prevents agonist-triggered FPR activation and internalization, decreases binding to and cell adhesion onto Vn and inhibits uPAR/VnR association. These findings uncover an inherent switch localized on Ser^90^ that potently affects uPAR activity.

This work is based on previous evidence showing that substitutions of Ser^90^ in the uPAR chemotactic sequence-derived peptides are critical to biological activity [28]. Our findings well fit with the previously reported ability of the synthetic peptides RERY and RPRY to inhibit or increase cell migration, respectively ([31], unpublished data). The analysis of the conformational preferences adopted by SuPAR chemotactic sequence of [32]–[34] shows that residue Ser^90^ is positioned in a critical “hinge”, which possibly influences the conformation of nearest residues (see Fig. 1A). A correlation between sequence features and hinges has been described by Flores et al., who found that some amino acid types such as serine are overrepresented in hinges, and much of this can be explained on the basis of physicochemical properties [39]. This information, together with the demonstration that Ser^90^ is functionally crucial can be considered a “proof of concept” and open the way for new molecular approaches to modulate uPAR signaling.

In the last decade, it has been suggested that unengaged uPAR may exist in a latent form and then may be subjected to a conformational change upon ligand binding [29]. Recently, Gårdsvoll and co-workers, have proposed a model in which the multidomain uPAR may reversibly acquire distinct conformational states that differently impact on its function. In the absence of uPA, a fraction of uPAR adopts an “*open conformation*”, which is unable to induce lamellipodia; uPA engagement or any perturbation of this equilibrium, shifts the structure of uPAR from an open to an intermediate, and then to a closed, but active, conformation [30]. For instance, Gårdsvoll et al., by covalently tethering domains DI and DIII via a non-natural interdomain disulfide bond, have generated a soluble form of uPAR which lacks conformational flexibility and results in a constitutively active uPAR which bypasses the regulatory role of uPA [40]. However, the equilibrium between the open and closed conformations may be sensitive to mutations, like the S90E and S90P presented in this paper. Recently, Xu et al., have analyzed the crystal structures of a stabilized, human uPAR (H47C/N259C) in its ligand-free form and in complex with ATF. They found that the domain boundary between uPAR DI–DII domains is more flexible than the DII–DIII domain boundary [41]. A likely possibility is that mutations of Ser^90^ may affect the orientation and position of the DI domain relative to DII–DIII domains and, as a consequence, affect receptor function. However, it still remains to be understood how DI domain moves, relative to DII and DIII domains, to give a closed, an intermediate or an open conformation. We have hypothesized that such movements might be associated to local conformational changes of the DI-DII linker domain. According to this model, cell surface uPAR^S90P^, which appears to retain an optimal Vn binding site, could assume the closed conformation of the uPA-binding cavity whereas the open conformation described by Gårdsvoll could be mimicked by the uPAR^S90E^ mutant that fails to bind to Vn.

In our studies, we have found that the Glu substitution of Ser^90^ modifies uPAR ability to interact with Vn. By an Ala-scan mutagenesis of the uPAR, Madsen *et al*. documented that Arg^91^, Tyr^92^ and Leu^93^ are crucial for uPAR binding to Vn, whereas Ser^90^, if substituted with an alanine residue neither impacts on cell morphology, nor modifies uPAR binding to Vn [24]. This suggests that Ser^90^ is not directly involved in binding to Vn (or is not a crucial determinant), but influences this interaction through local changes of conformation. Furthermore, the finding that uPAR^S90E^ exhibits a higher Kd*_app_* for ATF, suggests that the S90E substitution may affect the relative orientation of DI and DII-DIII domains. An interesting question relates to the receptor dimerization that has been documented to occur in response to ATF [42]. It will be interesting to assess if ATF still promotes dimerization of uPAR carrying S90E mutation.

Functionally, both S90P and S90E substitutions exert a profound impact on uPAR-dependent signal transduction. To our knowledge, this is the first dominant-negative variant of uPAR, which controls the activity of uPAR^wt^, when both receptors are co-expressed. In the highly invasive HT1080 fibrosarcoma cells, the expression of uPAR^S90E^ impairs uPAR-mediated signals, reducing cell wound repair and lung metastasis in nude mice. In these experiments, we have employed a pool of stable transfectants in which the average ratio between uPAR^S90E^ and uPAR^wt^ is approximately 2:1. Further experiments are needed to assess the minimal ratio required to negatively control uPAR function. In any event, together with blocking antibodies, inhibitory peptides, interfering RNAs, this could be a novel approach to study uPAR function.

We and others have documented that the uPAR_88–92_ sequence is an agonist of FPR, FPR Like-1 and FPR Like-2, also in the form of an isolated peptide SRSRY, [20]–[21], [43]. FPRs have been detected in cells of haematopoietic and non-haematopoietic origin, such as lung epithelial, hepatocytes, dendritic cells, bone marrow-derived mesenchymal stem and endothelial cells [22], [44]–[47]. Their ability to bind different and apparently unrelated ligands [48] raises the possibility that FPR agonists/inverse agonists or antagonists could be considered as novel anti-inflammatory therapeutics for the treatment of a variety of clinical conditions, including neurodegenerative disease and stroke [48]. Here, we show that uPAR^S90P^ seems to behave as a super agonist as it activates FPR more efficiently than uPAR^wt^, leading to an increase of random and directional cell migration, long-lasting AKT phosphorylation and uPAR/integrin association.

In the last decade, numerous efforts attempting to develop new and specific pharmaceuticals targeting the function of uPAR for the treatment of cancer have been done [4], [49]. Disappointingly, none of these approaches has so far reached clinical testing. More recently, several approaches targeting uPAR interactions upon uPA binding (*e.g*. uPAR/integrins or uPAR/FPRs interactions) have been generated and novel uPAR targeted proof-of-principle approaches have been described [4]. In this respect, we have already generated synthetic peptides mimicking uPAR^S90P^ and uPAR^S90E^ mutants and showed that a peptide containing the Arg-Glu-Arg central core prevents malignant dissemination in nude mouse [28]. This study confirms that Ser^90^ with its surrounding chemotactic sequence is crucial to uPAR function and provides further support to the generation of uPAR_88–92_-derived peptides, as drugs targeting uPAR function.

## Materials and Methods

### Plasmids

The expression vector pcDNA3-uPAR was constructed by inserting the 1027 bp *Eco*RI-*Eco*RI fragment from pBluescript II SK, containing the whole uPAR-cDNA [18]. uPAR cDNAs encoding uPAR variants carrying S90E or S90P substitutions were generated with a site-specific mutagenesis kit (Stratagene), according to the manufacturer's instruction, using the following primers:

uPAR^S90E^ cDNA r, 5′-TCACCTATTCCCGAGAACGTTACCTCGAATG-3′,

uPAR^S90E^ cDNA f, 3′-CATTCGAGGTAACGTTCTCGGGAATAGGTGA-5;

uPAR^S90P^ cDNA r, 5′-TCACCTATTCCCGACCCCGTTACCTCGAATG-3′,

uPAR^S90P^ cDNA f, 3′-CATTCGAGGTAACGGGGTCGGGAATAGGTGA-5′.

All mutations were confirmed by DNA sequencing.

### Cell culture and generation of stable transfectants

Human embryonic kidney (HEK) 293 cells, and human fibrosarcoma HT1080 cell lines were grown in DMEM supplemented with 10%FBS, 100 IU/ml penicillin and 50 µg/ml streptomycin.

The expression vectors pcDNA3-uPAR^wt^, pcDNA3-uPAR^S90P^ and pcDNA3-uPAR^S90E^, encoding uPAR^wt^, uPAR^S90P^ and uPAR^S90E^, respectively, were transfected into 293 or HT1080 cells using FuGENE 6 transfection reagent, according to the manufacturer's specifications (Roche Applied Science). Stable cell lines were established by selection with G418 (800 μg/ml). 293 transfectants were cloned by limiting dilution at 0.5 cells/well.

### Western blot

When specified, cells were exposed to increasing concentrations of ATF at 37°C for the indicate times. In all cases, detached cells (1×10^6^/sample) were lysed in RIPA buffer (10 mM Tris pH 7.5, 140 mM NaCl, 0.1%SDS, 1% Triton X-100, 0.5% NP40) containing protease inhibitor mixture. Protein content of cell lysates was measured by a colorimetric assay (BioRad). Twenty-five or five hundred nanograms of proteins were separated on 10% SDS-PAGE and transferred onto a polyvinylidene fluoride membrane. The membranes were blocked with 5% non-fat dry milk and probed with 1 µg/ml R4 anti-uPAR monoclonal antibody (mAb) recognizing uPAR DIII domain, or VNR139 anti-αv chain mAb (Life Technologies), or anti-phosphoAKT (S473) polyclonal antibodies (Ab)s, or total AKT mAb, both purchased by R&D Systems. In all cases, washed filters were incubated with horseradish peroxidase-conjugated anti-mouse or anti-rabbit antibody and detected by ECL (Amersham). Densitometry was performed by NIH Image 1.62 software (Bethesda, MD).

### Radio-iodination and binding assay

5 µg ATF(American Diagnostica) or Vn (Promega) were radio iodinated with Na^125^I using IODO-BEADS (Pierce) in 0.1 M sodium phosphate, 0.15 M NaCl pH 7.2 for 10 minutes at 25°C in a final volume of 100 µl, as previously described [18]. The radio-labeled proteins were purified from unbound iodide by Sephadex G-25 chromatography and the resulting specific activity was approximately 5 µCi/µg. Binding assays were performed on adherent cells (2×10^5^cells/well) grown in 24-multiwell flat bottom plates. Cells were incubated for 3 hours at 4°C with 1 nM ^125^I-ATF or 10 nM ^125^I-Vn diluted in binding buffer (DMEM, 1 mg/ml BSA, 10 mM Hepes pH 7.4), in the absence or in the presence of increasing unlabeled ATF or Vn, respectively. Some experiments was performed on 293/uPAR^wt-3^ cell-pre-exposed to 2.5 µg/ml anti-RGD-binding site P1F6 mAb (Chemicon) for 1 hour at room temperature. A subset of experiments was performed analyzing cells (2×10^6^/sample) in suspension. In all cases, at the end of incubation, cells were washed three times with binding buffer and the surface-associated proteins were recovered by treating cells with an acidic wash (50 mM glycine-HCl buffer pH 3.0, containing 0.1 M NaCl) for 10 minutes at room temperature. Acid washes were collected and counted in a gamma counter. Each experiment was carried out in duplicate and the results were plotted as mean cpm ± SD.

### Flow cytometry

Cells (0.5×10^6^ cells/sample) were detached using 200 mg/L EDTA, 500 mg/L trypsin (Cambrex) and incubated with phosphate-buffered saline (PBS), non immune serum or 2 µg/ml R4 anti-uPAR mAb for 30 minutes at 4°C. After extensive washing with PBS, cells were incubated with 1∶200 Alexa 488-conjugated F(ab')2 fragment of rabbit anti-mouse IgG (Molecular Probes) for 30 min in the dark and, finally, re-suspended in 0.5 ml PBS. Samples were analysed by flow cytometry using a FACS Aria II and DIVA software (Becton & Dickinson).

### Immunocytochemistry

Cells (∼ 2×10^4^) were seeded on glass coverslips and cultured for 24 hours in DMEM plus 10% FBS. Briefly, slides were washed with PBS and fixed with 2.5% formaldehyde in PBS for 10 minutes at 4°C, then incubated overnight at 4°C with 2 µg/ml R4 anti-uPAR mAb or diluents. After several washes in PBS, 1∶200 diluted biotinylated goat anti-mouse immunoglobulins were applied to slides at 23°C for 60 minutes, as previously described [50]. Thereafter, cells were incubated with streptavidin-biotinylated horseradish peroxidase complex for additional 30 minutes and the peroxidase-dependent staining was developed by diaminobenzidine (Vector Lab). Slides were counterstained with Mayer's haematoxylin.

### Fluorescence microscopy

Cells grown on glass slides to semi-confluence were exposed to 10 nM *N*-formyl-Nle-or Leu-Phe-Nle-Tyr-Lys-fluorescein (Molecular Probes), diluted in serum-free DMEM for 30 minutes at 37°C as described [27]. After several washes in PBS, coverslips were mounted using 20% (w/v) Mowiol. Cells were visualized with a Zeiss 510 META-LSM microscope, and z-series with 0.25 µm intervals were collected. To analyze cytoskeleton, cells grown on glass slides to semi-confluence, were fixed with 2.5% formaldehyde, permeabilized with 0.1% Triton X-100 for 10 minutes at 4°C, and then incubated with 0.1 µg/ml rhodamine-conjugated phalloidin (Sigma-Aldrich) for 40 minutes, as previously described [21].

### Co-immunoprecipitation of αv/uPAR complexes

Cells exposed for 1 hour to 10 nM or 100 nM ATF or diluents at 37°C were lysed in RIPA buffer. Extracts (400 µg/sample) were incubated overnight at 4°C with 5 µg/ml anti-αv Ab (Chemicon) as described [17]. Proteins co-purified with αv were recovered by Protein G-Sepharose and analyzed by a 10% SDS-PAGE followed by Western blot with 2 µg/ml R4 anti-uPAR mAb or 1 µg/ml VNR147 anti-αv mAb (Chemicon).

### Cell adhesion assay

Cell adhesion assays were performed using 24-well tissue culture plates coated with 5 µg/ml Vn, 5 µg/ml fibronectin (Fn) or heat-denatured BSA diluted in PBS and incubated overnight at 4°C. The plates were rinsed with PBS, incubated for 1hour at 23°C with 1% BSA in PBS, and rinsed again. Cells (2×10^5^cells/well) were plated in each coated well and incubated for 2 hours at 37°C, 5% CO_2_, in serum-free DMEM, in the presence or in the absence of 5 µg/ml 399 anti-uPAR Ab. After three washes with PBS, adherent cells were detached and counted.

### Time-lapse imaging of cell migration

Live-cell imaging was performed at 37°C, 5% CO2 using an inverted phase-contrast microscope (Axiovert 200, Zeiss) equipped with a motorized stage and an incubation chamber. For single cell motility, 5×10^3^ cells/sample were plated in growth medium. After 24 hours, images were acquired for 70 minutes (1 frame every 15 sec). Cell migration speed, straight distance and tortuosity were quantified using the Axiovision 4.8 software (Carl Zeiss). In each experiment, at least 10 randomly chosen cells were tracked and their average straight distance and tortuosity (the last has been calculated as the total path length of the cells migration divided by the displacement between the initial and final cell positions) throughout the experiment were calculated. For wound-healing assays, confluent cells grown in a 24 multi-well plate were wounded with a sterile pipette tip and exposed to 10% FBS-DMEM. One field/dish including the scratched path was selected and scanned sequentially every 30 minutes for 20 hours. The extent of wounded areas was evaluated by the Axiovision 4.8 software.

### Cell migration and invasion assays

Chemotaxis assays were performed in Boyden chambers, using 8µm pore size PVPF-filters (Nucleopore) as previously described [21]. Briefly, 1×10^5^ viable cells were seeded in each upper chamber in serum-free DMEM. The lower chamber was filled with DMEM containing 10 nM ATF, 10 nM fMLF or 10% FBS as a source of chemoattractants. Cells were allowed to migrate for 4 hours at 37°C, 5% CO_2._ For invasion assays, 3×10^4^ cells/chamber were allowed to invade matrigel for 18 hours at 37°C, 5% CO_2_ using filters coated with 25 µg matrigel (BD Biosciences) and 10 nM ATF or 10% FBS in DMEM as chemoattractants [50]. In all cases, at the end of the assay, cells on the lower filter surface were fixed with ethanol, stained with haematoxylin and 10 random fields/filter were counted at 200x magnification. The arbitrary value of 100% was given to the basal cell migration or invasion, assessed in the absence of chemoattractant. In all cases, the experiments were performed at least twice in triplicate, and the results, expressed as percentage of the basal cell migration/invasion.

### Cell proliferation assay

Cells (1.5×10^4^cells/well) were grown in 24-multiwell flat bottom plates in 10% FBS-DMEM. At the indicated times, non-adherent cells were removed, while adherent cells were detached and counted.

### *In vivo* metastasis assay

Thirty 21 to 23gr, six-eight week old, CD1 female nude mice were maintained in a germ-free environment. Housing and handling of mice were in accordance with institutional guidelines complying with national and international laws and policies. Mice (6 animals/group) received an injection of wild type or transfected HT1080 cells (1.5×10^6^ cells as a single-cell suspension in 100 µl of sterile PBS, 98% viability) in the tail vein. After 22 days, mice were weighted, and sacrificed by cervical dislocation. Lungs were removed and immediately fixed in buffered 4% formaldehyde for the histological analysis. The extent of normal and tumor lung parenchyma was measured in three/five not serial haematoxilin-stained sections examined blindly under light microscope by two independent observers at 5x magnification. Morphometric analysis was performed using the Axiovision 4.8 software. Data were expressed as percentage of total area measured in each tissue section.

### Statistical analysis

The results are expressed as the mean ± SD of the number of the indicated determinations. The statistical tests used to assess the differences were determined by Student's t-test (*p*<0.001 was accepted as significant).

### Ethics statement

The research work with mouse model has been approved by Institutional Ethical Committee of the National Cancer Institute, Naples (protocol n. 09, December 20, 2010).

## Supporting Information

Figure S1 **Basal cell migration of uPAR expressing 293 cells.** Phase-contrast time-lapse of 293/uPAR^wt-3^ cells were acquired for 70 minutes (1 frame every 15 s). The movie includes overlay tracking of 9 randomly chosen cells.
(MOV)

Figure S2 **Basal cell migration of 293 cells expressing uPAR^S90P^.** Phase-contrast time-lapse of 293/uPAR^S90P-G^ cells were acquired for 70 minutes (1 frame every 15 s). The movie includes overlay tracking of 9 randomly chosen cells.
(MOV)

Figure S3 **Basal cell migration of of 293 cells expressing uPAR^S90E^.** Phase-contrast time-lapse of 293/uPAR^S90E-3^ cells were acquired for 70 minutes (1 frame every 15 s). The movie includes overlay tracking of 12 randomly chosen cells.
(MOV)

Figure S4 **Wound healing assay of HT1080 cells.** Wound healing of uPAR bearing HT1080 cells kept in growth medium at 37°C in a 5% CO_2_ of a Zeiss inverted microscope equipped with a motorized stage. One field which includes the scratched path from each dish was selected and scanned sequentially every 30 minutes for 20 hours.
(MOV)

Figure S5 **Wound healing assay of mock transfected HT1080 cells.** Wound healing of HT1080/mock cells kept in growth medium at 37°C in a 5% CO_2_ of a Zeiss inverted microscope equipped with a motorized stage. One field which includes the scratched path from each dish was selected and scanned sequentially every 30 minutes for 20 hours.
(MOV)

Figure S6 **Wound healing assay of HT1080 cells overexpressing uPAR.** Wound healing of HT1080/uPAR^S90^cells kept in growth medium at 37°C in a 5% CO_2_ of a Zeiss inverted microscope equipped with a motorized stage. One field which includes the scratched path from each dish was selected and scanned sequentially every 30 minutes for 20 hours.
(MOV)

Figure S7 **Wound healing assay of HT1080 cells co-expressing wild type and S90E mutated form of uPAR.** Wound healing of HT1080/uPAR^S90E^ cells kept in growth medium at 37°C in a 5% CO_2_ of a Zeiss inverted microscope equipped with a motorized stage. One field which includes the scratched path from each dish was selected and scanned sequentially every 30 minutes for 20 hours.
(MOV)

Figure S8 **Wound healing assay of HT1080 cells co-expressing wild type and S90P mutated form of uPAR.** Wound healing of HT1080/uPAR^S90P^ cells kept in growth medium at 37°C in a 5% CO_2_ of a Zeiss inverted microscope equipped with a motorized stage. One field which includes the scratched path from each dish was selected and scanned sequentially every 30 minutes for 20 hours.
(MOV)
